# Supplementary material for: Understanding opposing predictions of Prochlorococcus in a changing climate
Source: Nat Commun. 2023 Mar 15;14:1445. doi: 10.1038/s41467-023-36928-9 (PMC10017810; doi:10.1038/s41467-023-36928-9)
Supplement: Supplementary file 1 — Supplementary Information [file 41467_2023_36928_MOESM1_ESM.pdf]

Supplementary Information for: Understanding Opposing Predictions of *Prochlorococcus* in a Changing Climate

Vincent Bian[1], Merrick Cai[2], and Christopher L. Follett[3*]

[1]Department of Physics, Massachusetts Institute of Technology, Cambridge, Massachusetts, USA.
[2]Department of Mathematics, Massachusetts Institute of Technology, Cambridge, Massachusetts, USA.
[3]Department of Earth, Atmospheric and Planetary Sciences, Massachusetts Institute of Technology, Cambridge, Massachusetts, USA.
[*]Corresponding Author: Christopher L. Follett, 77 Massachusetts Ave., Bldg. 54-1511a, Cambridge, MA, 02139. Email: follett@mit.edu

March 10, 2023

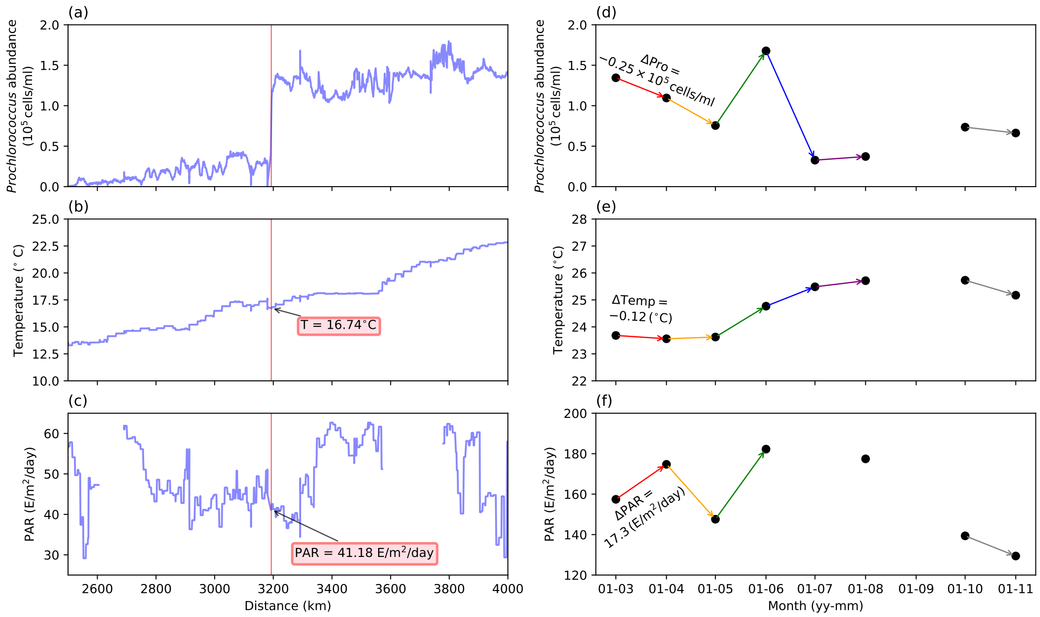

Supplementary Figure 1: Methods for Analyzing Spatial and Temporal Change: (left column) Sharp changes in the concentration of *Prochlorococcus* cells (panel a) are determined using a wavelet approach (see Methods) and then co-localized with temperature (panel b) and Photosynthetically Active Radiation (panel c). (right column) Temporal fluctuations in *Prochlorococcus* concentrations (panel d) from Station ALOHA are calculated as month to month differences and compared with co-localized changes in temperature (panel e) and PAR (panel f).

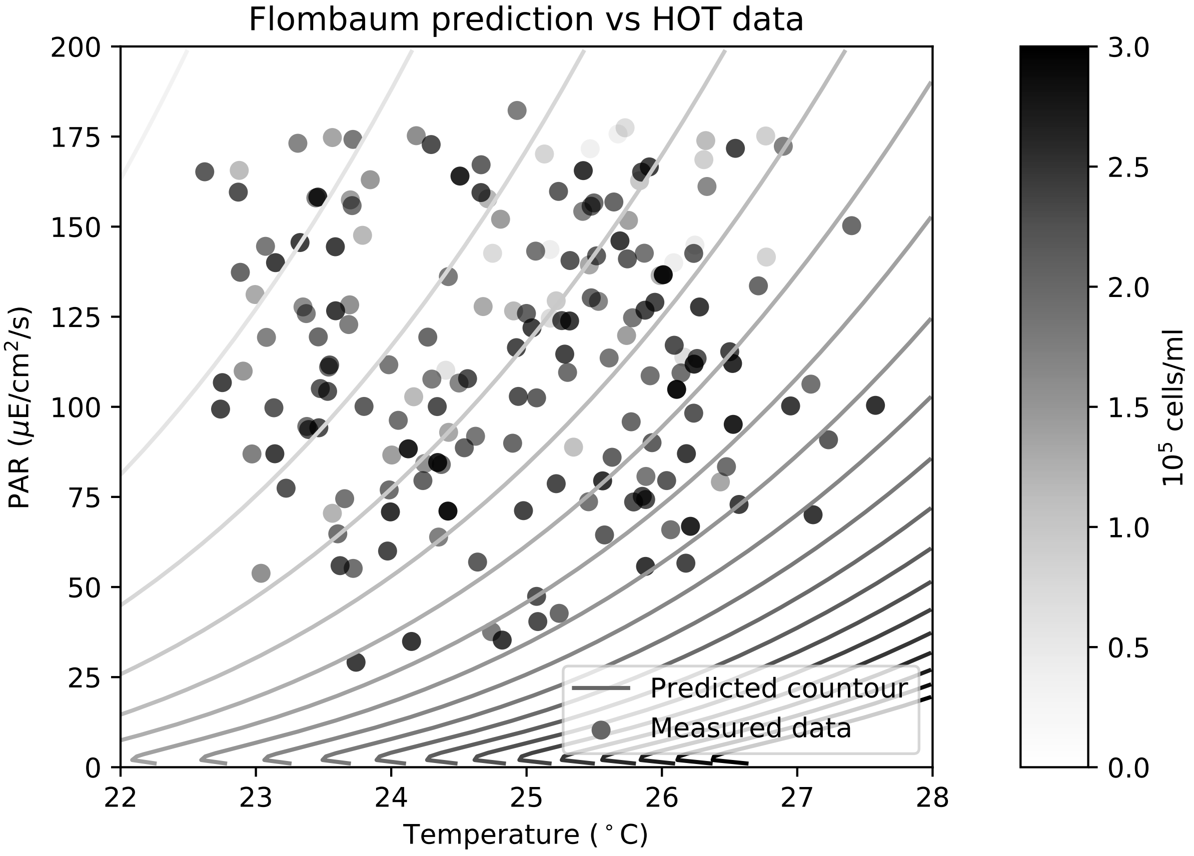

Supplementary Figure 2: *Prochlorococcus* abundance data from station ALOHA (< 50 meters depth) is plotted vs. PAR and Temperature. Overlaid are the predictions from the Flombaum model as shaded contours.

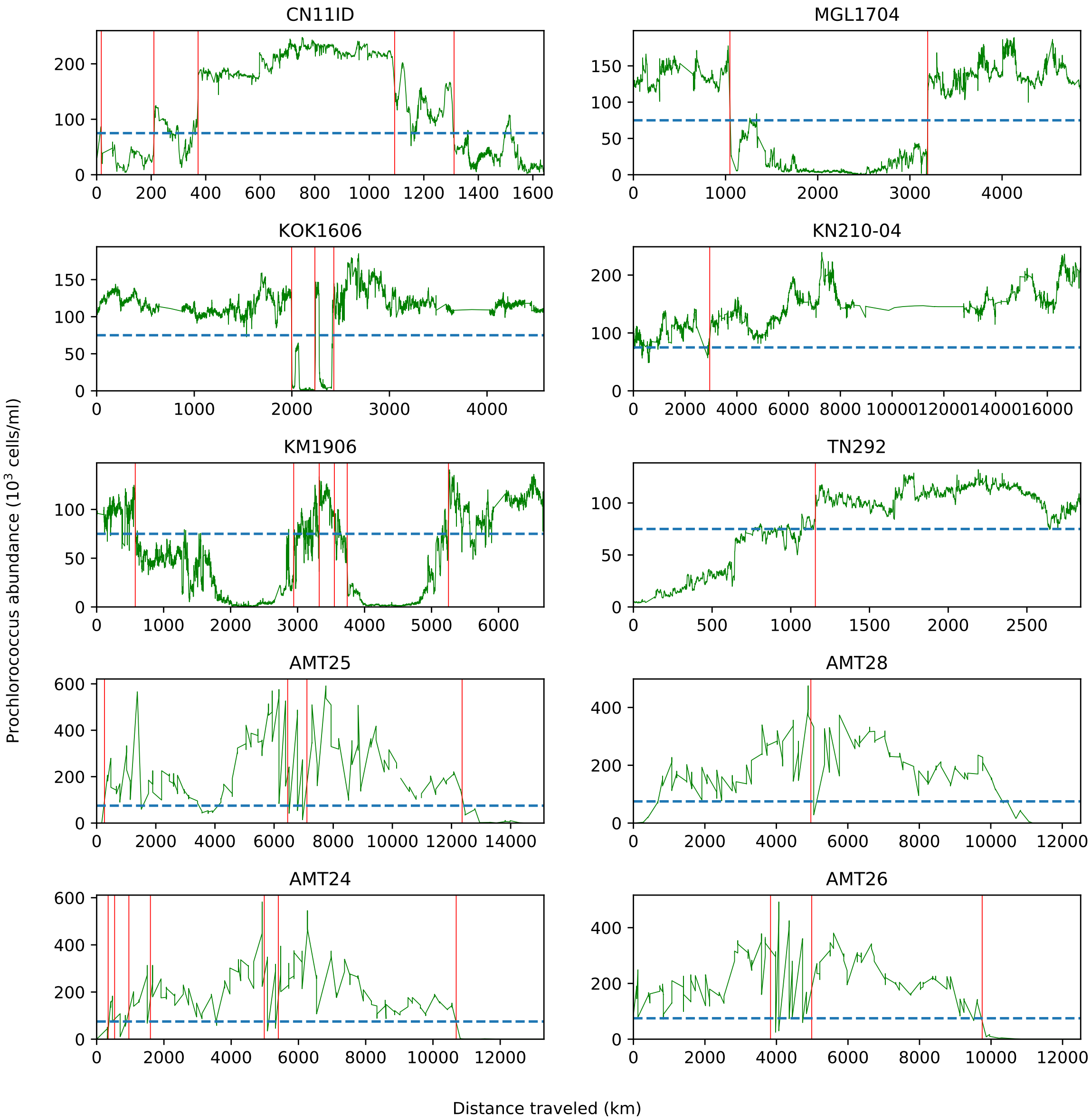

Supplementary Figure 3: A surface *Prochlorococcus* concentration vs. distance plot for every cruise containing a detected transition (SeaFlow and AMT transects) which passes through the 75,000 cells/ml concentration value. Transitions are shown in red and the concentration threshold as a blue dashed line. Due to temperature/PAR measurements being unavailable for some of these data points, this plot shows more crashes than Figure 4.

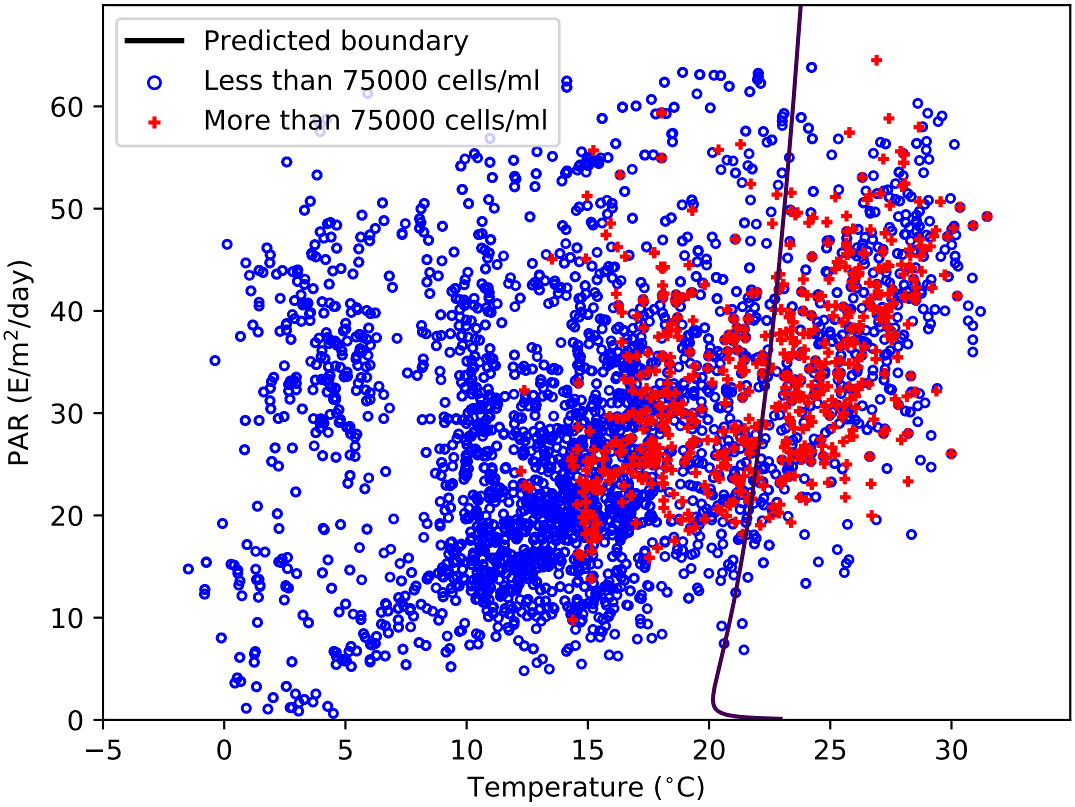

Supplementary Figure 4: The Flombaum data set is plotted vs. PAR and temperature. Red denotes that the sample contained less than $75,000$ *Prochlorococcus* cells/mL and blue more than $75,000$ cells/mL. If the Flombaum model were exactly accurate, all the red points would be to the right of the curve and all the blue points would be to the left.

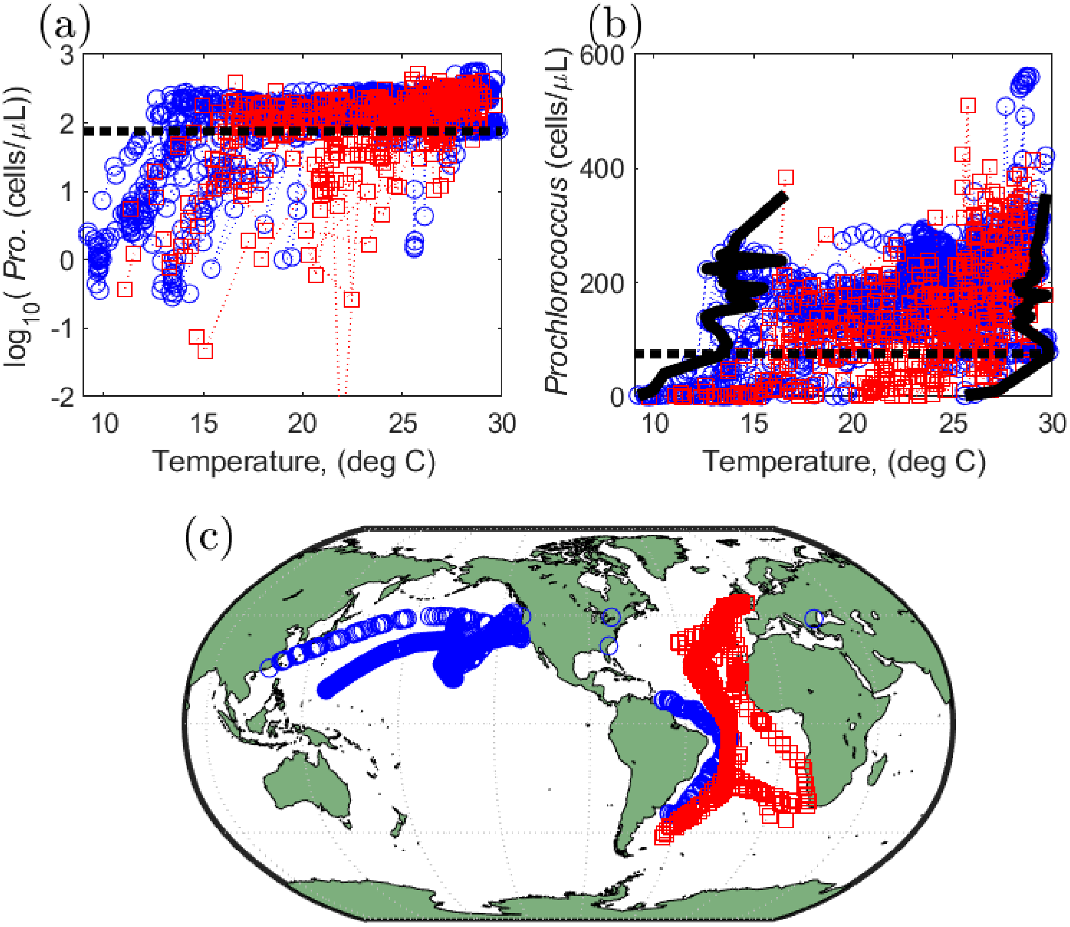

Supplementary Figure 5: AMT and SeaFlow Data vs. Temperature: Surface data from SeaFlow (blue circles, in $\sim$ 200 measurement bins), and the AMT program (red squares, median measurement from upper 50 meters) is plotted. (a) Log of *Prochlorococcus* abundance is plotted vs. surface temperature. Horizontal dashed line is the 75 cells/μL threshold. (b) *Prochlorococcus* abundance is plotted vs. surface temperature. Horizontal dashed line is the 75 cells/μL threshold. Solid black lines are the estimated range of the observations taken from consecutive groups (100 samples) of data taken from lowest to highest abundance. (c) A map of the locations of all samples shown in panels a-b.

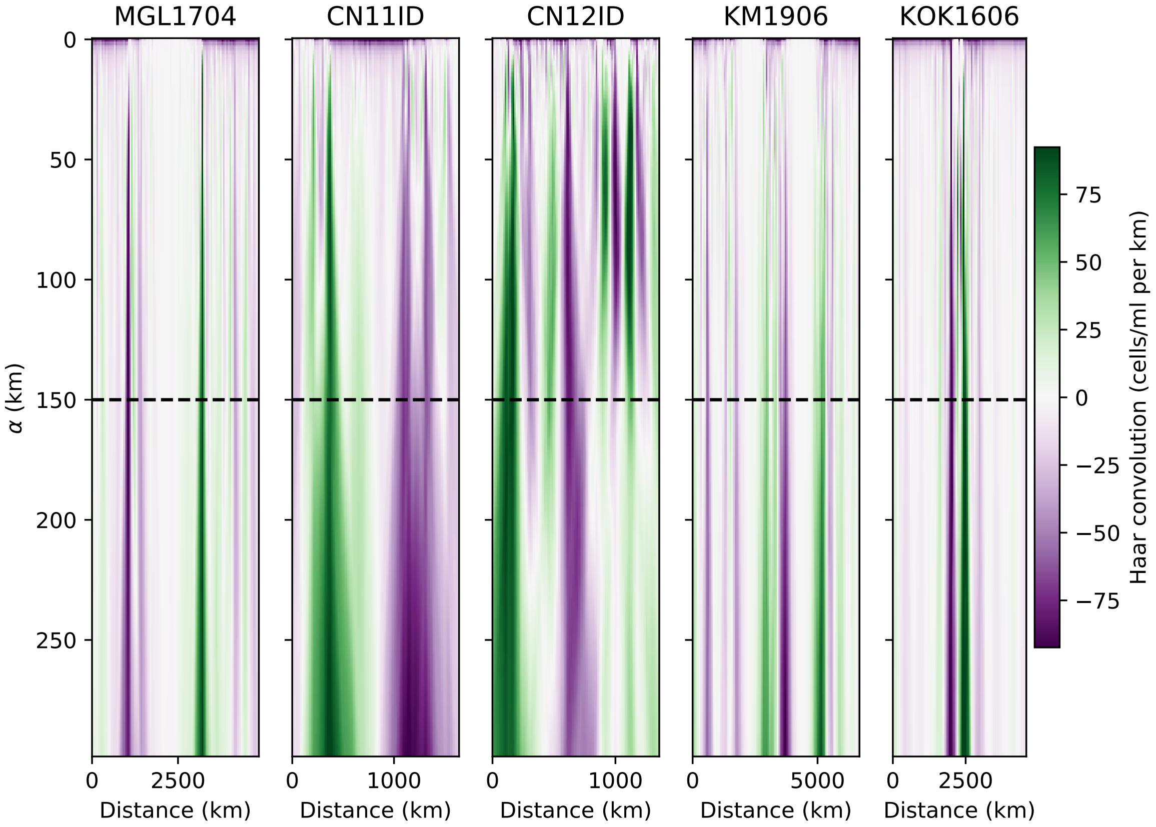

Supplementary Figure 6: The output of the Haar wavelet transform for 5 selected cruises. The scale break occurs near $\alpha = 150\,\text{km}$, and is particularly apparent in the graphs of CN11ID and CN12ID.

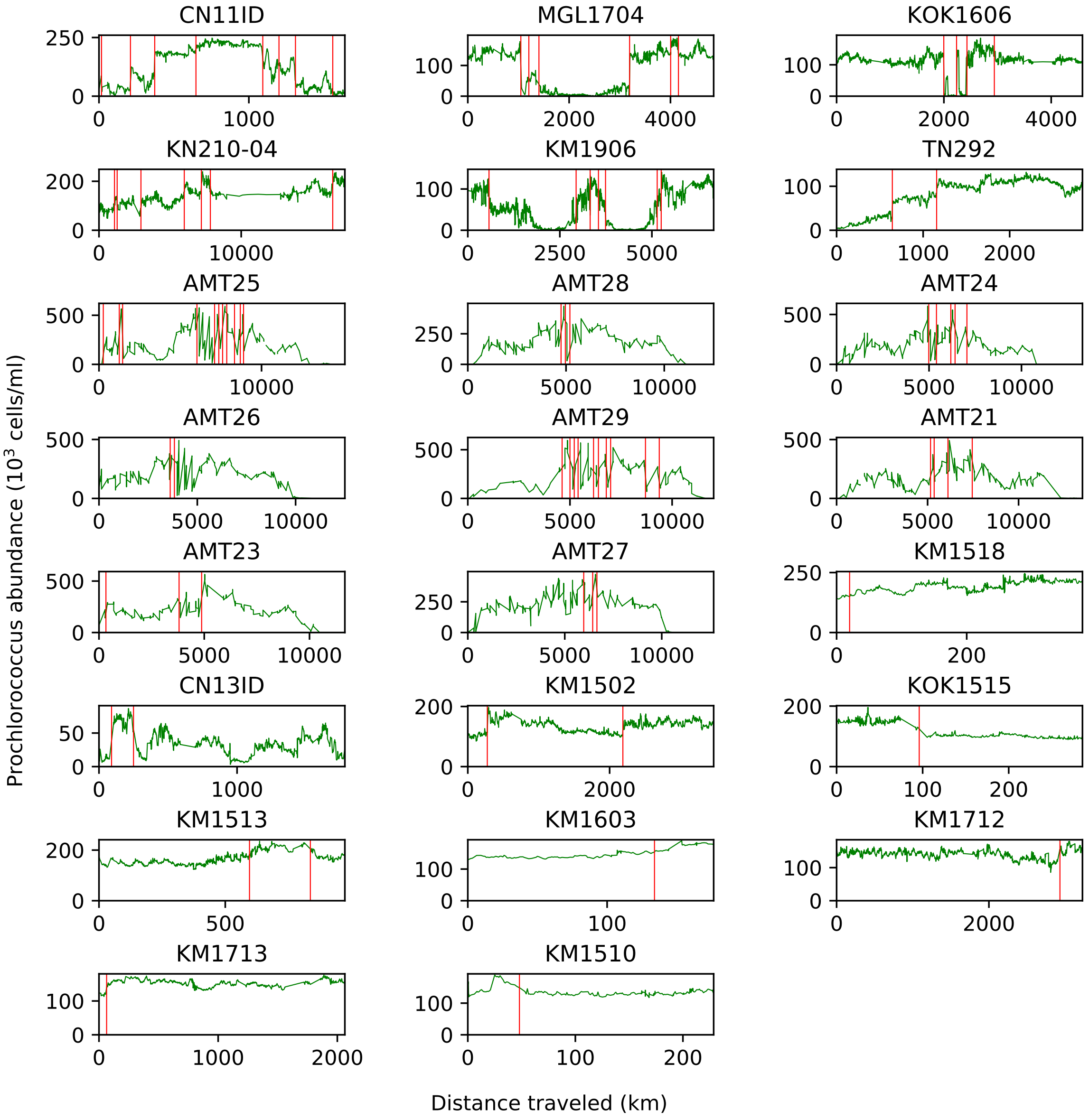

Supplementary Figure 7: A surface *Prochlorococcus* concentration vs. distance plot for every cruise containing a detected transition (SeaFlow and AMT transects). Detected transitions are shown by the vertical red lines. The subset of these transitions passing through 75,000 cells/ml is shown in Supplementary Figure 3.

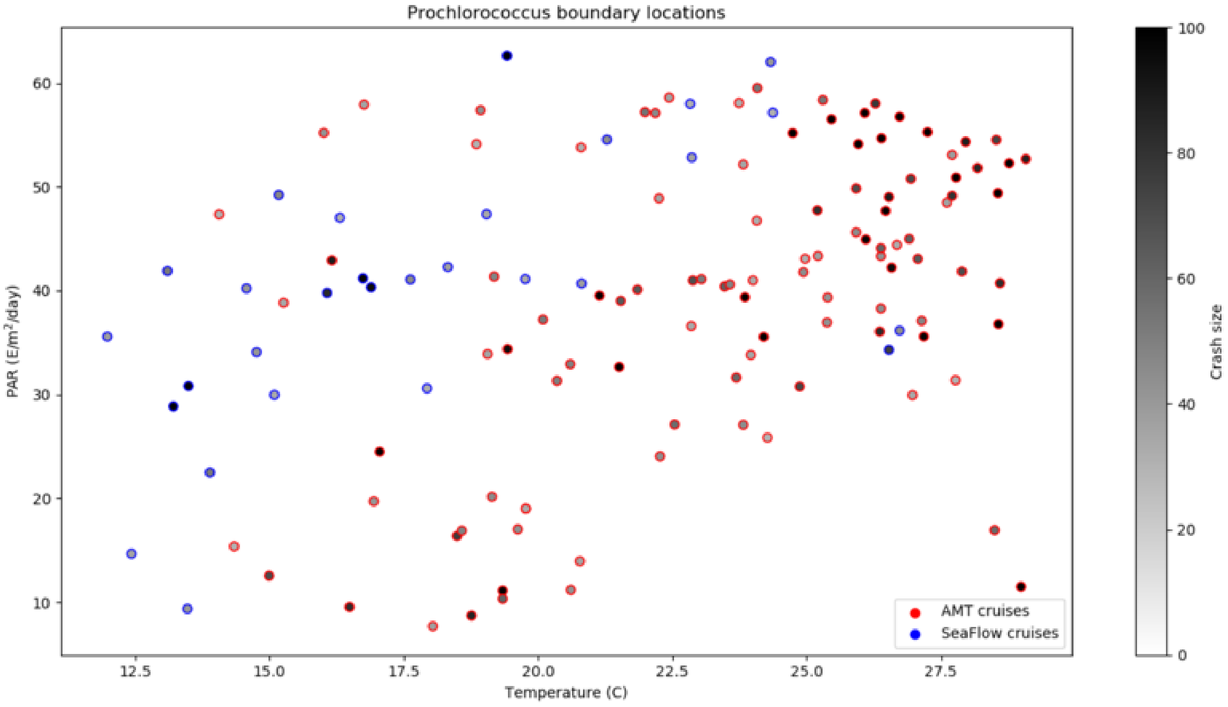

Supplementary Figure 8: All transitions found in the SeaFlow (blue) and AMT datasets (red) plotted vs. PAR and temperature. The shading of the circle represents the magnitude of the population shift in units of 10^3 cells/ml. The subset of transitions which cross the 75,000 cells/mL concentration value are shown in Figure 4.

	Residuals of Flombaum Model	Residuals of Two-State Model
Log space, R	0.663 ± 0.005	0.644 ± 0.006
Log space, μ	-0.811 ± 0.016	-0.816 ± 0.017
Log space, σ	1.765 ± 0.012	1.890 ± 0.014
Linear space, R	$0.544 \pm .009$	$0.390 \pm .005$
Linear space, μ	5531 ± 391	-320 ± 428
Linear space, σ	42513 ± 764	46620 ± 804

Supplementary Table 1: Residuals of the Flombaum vs. Two-state model in log and linear space.

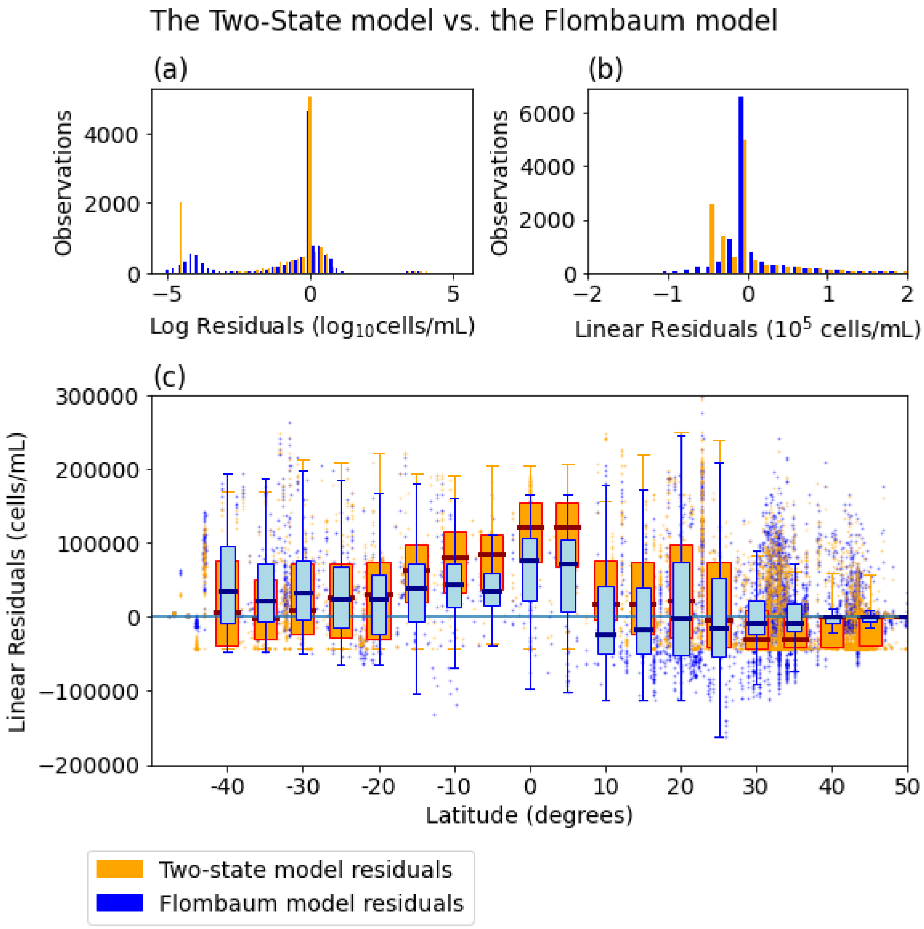

Supplementary Figure 9: The residuals of the Flombaum model are compared with those of the two-state model for the surface ocean. The two-state model is denoted by red/orange and the Flombaum model by blue (the color scheme applies for both the scatter points as well as the box-and-whisker plots). In all three of the plots there are $n = 11930$ data points which contain the measured *Prochlorococcus* abundance, the temperature of the surface ocean, and the PAR. (a) A histogram of the residuals of both the Flombaum and constant models in logarithmic space. (b) A histogram of the residuals of both models in linear space. (c) The linear residuals of both models are plotted vs. latitude. The box-and-whisker plots are given every 5 degrees of latitude from 40°S to 45°N, one for the residuals of the Flombaum model, and one for the residuals of the two-state model. Each box-and-whisker plot represents the boxes by the first quartile, median, and third quartile values, and the whiskers are 1.5 relative to the median, for residuals of data points taken at latitudes between 5° north or south of the latitude for which it appears in the figure above. The horizontal blue line is at 0, which represents the data points for which (either of) the models' predictions agree with the measured abundance.

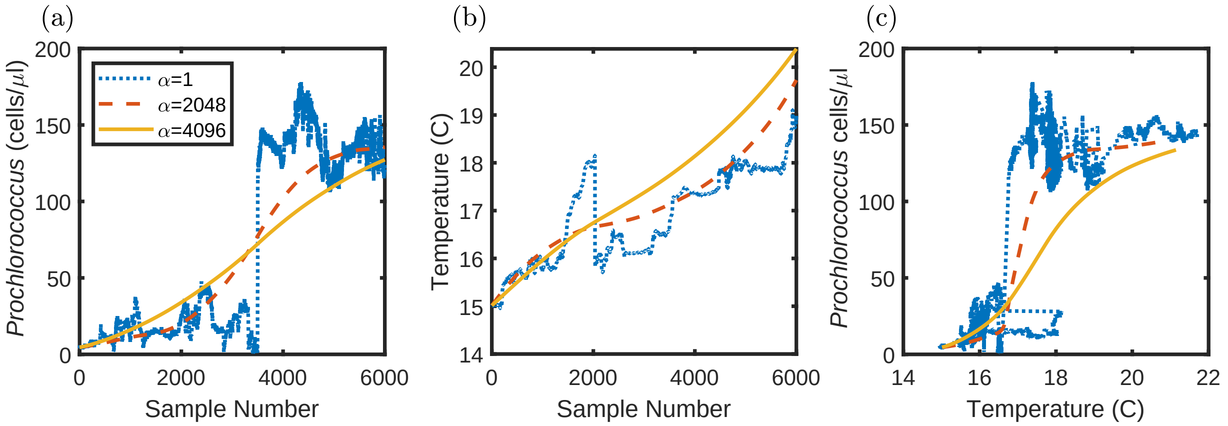

Supplementary Figure 10: Sharp Transitions with Spatial Scale: The section of cruise MGL1704 from approximately 2500 to 3500 km (see Supplementary Figure 3) is reconstructed from the spatial derivatives estimated as a function of wavelet scale, α, in units of sample number. Here, $\alpha = 1$ corresponds to $\sim< 1$ km, $\alpha = 2048$ to $\sim$ 1000 km and $\alpha = 4096$ to $\sim$ 2000 km. (a) The reconstruction is plotted along the cruise track. (b) The same reconstruction as in (a) except with surface temperature values. (c) Reconstructed *Prochlorococcus* abundance vs. reconstructed temperature.
