# Supplementary material for: Understanding opposing predictions of Prochlorococcus in a changing climate
Source: Nat Commun. 2023 Mar 15;14:1445. doi: 10.1038/s41467-023-36928-9 (PMC10017810; doi:10.1038/s41467-023-36928-9)
Supplement: Supplementary file 3 — Description of Additional Supplementary Files [file 41467_2023_36928_MOESM3_ESM.pdf]

Description of Additional Supplementary Files

File name: Supplementary Code
Description: This folder contains all the files required to do the central analyses in the main text. A readme.txt is included with additional details.
