# Supplementary figures and images for: Understanding opposing predictions of Prochlorococcus in a changing climate
Source: Nat Commun. 2023 Mar 15;14:1445. doi: 10.1038/s41467-023-36928-9 (PMC10017810; doi:10.1038/s41467-023-36928-9)

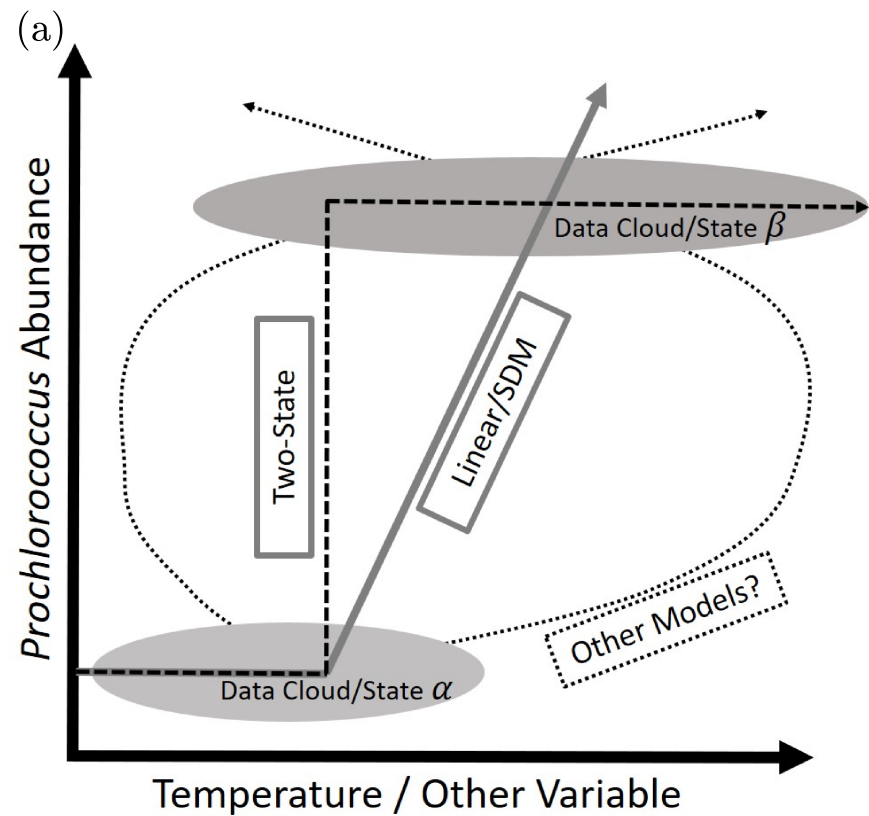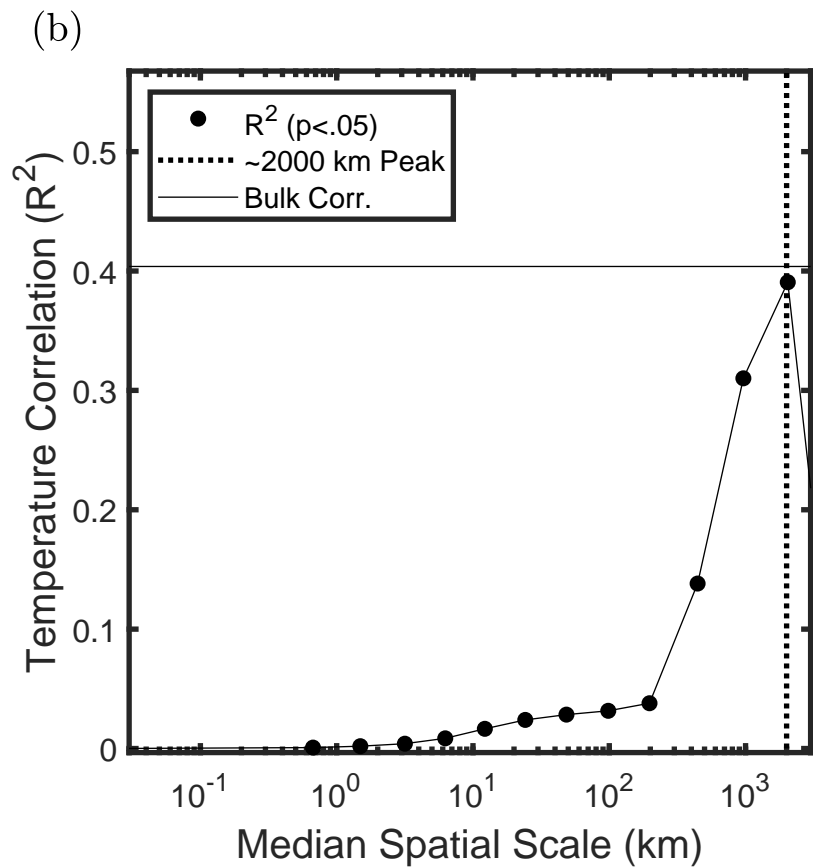

Supplement: Supplementary file 4 — Supplementary Code [file 41467_2023_36928_MOESM4_ESM.zip › Supplemental Code/AnalysisForFig5/Figure5.pdf]

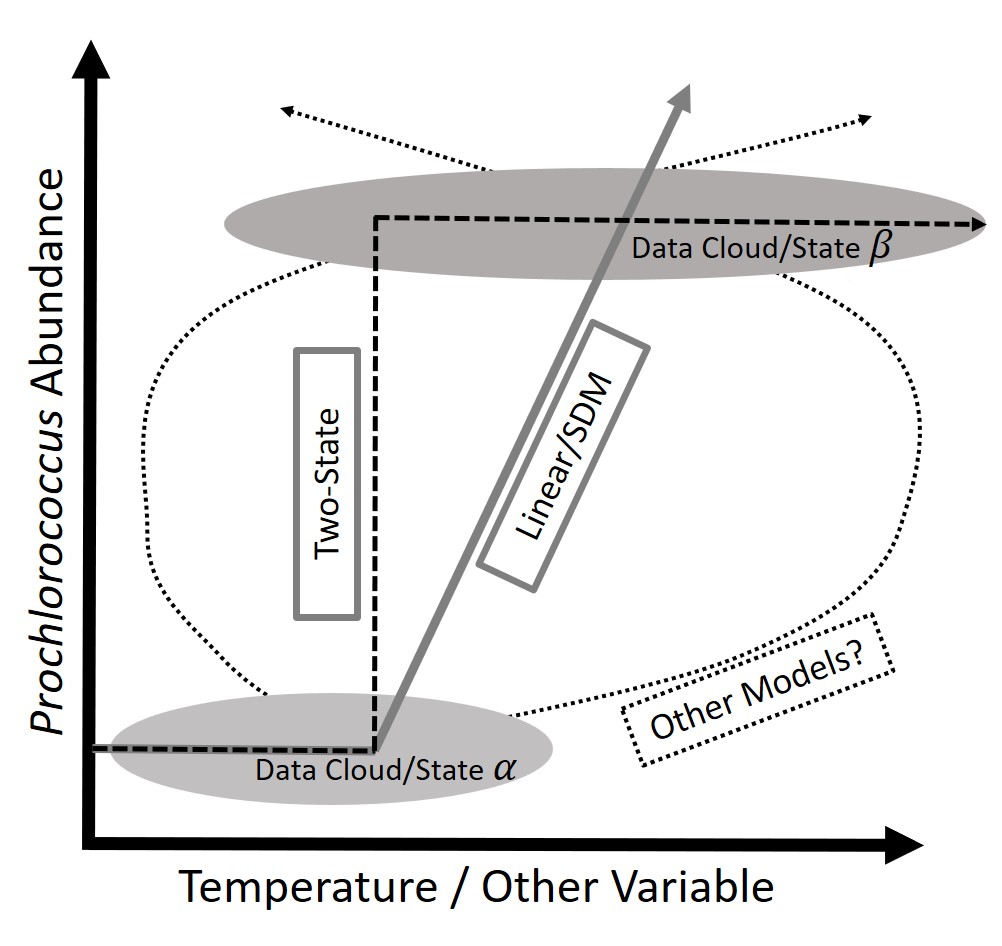

Supplement: Supplementary file 4 — Supplementary Code [file 41467_2023_36928_MOESM4_ESM.zip › Supplemental Code/AnalysisForFig5/twostateschematic.jpg]
