# Supplementary material for: Understanding opposing predictions of Prochlorococcus in a changing climate
Source: Nat Commun. 2023 Mar 15;14:1445. doi: 10.1038/s41467-023-36928-9 (PMC10017810; doi:10.1038/s41467-023-36928-9)
Supplement: Supplementary file 5 — Reporting Summary [file 41467_2023_36928_MOESM5_ESM.pdf]

nature portfolio

Corresponding author(s): Christopher L. Follett

Last updated by author(s): February 7, 2023

Reporting Summary

Nature Portfolio wishes to improve the reproducibility of the work that we publish. This form provides structure for consistency and transparency in reporting. For further information on Nature Portfolio policies, see our Editorial Policies and the Editorial Policy Checklist.

Statistics

For all statistical analyses, confirm that the following items are present in the figure legend, table legend, main text, or Methods section.

n/a	Confirmed	
☐	☒	The exact sample size (n) for each experimental group/condition, given as a discrete number and unit of measurement
☒	☐	A statement on whether measurements were taken from distinct samples or whether the same sample was measured repeatedly
☐	☒	The statistical test(s) used AND whether they are one- or two-sided<br>*Only common tests should be described solely by name; describe more complex techniques in the Methods section.*
☐	☒	A description of all covariates tested
☐	☒	A description of any assumptions or corrections, such as tests of normality and adjustment for multiple comparisons
☐	☒	A full description of the statistical parameters including central tendency (e.g. means) or other basic estimates (e.g. regression coefficient) AND variation (e.g. standard deviation) or associated estimates of uncertainty (e.g. confidence intervals)
☐	☒	For null hypothesis testing, the test statistic (e.g. F, t, r) with confidence intervals, effect sizes, degrees of freedom and P value noted<br>*Give P values as exact values whenever suitable.*
☐	☒	For Bayesian analysis, information on the choice of priors and Markov chain Monte Carlo settings
☒	☐	For hierarchical and complex designs, identification of the appropriate level for tests and full reporting of outcomes
☐	☒	Estimates of effect sizes (e.g. Cohen's d, Pearson's r), indicating how they were calculated

Our web collection on statistics for biologists contains articles on many of the points above.

Software and code

Policy information about availability of computer code

Data collection	Tools from the Simons Foundation CMAP (http://www.simonscmap.com, pycmap} API available at https://github.com/simonscmap/pycmap/archive/master.zip), were used to collect the data from previously published sources.
Data analysis	Data was analyzed using scripts developed in MATLAB (R2021a) and Python with additional tools from the Simons CMAP project (pycmap API https://github.com/simonscmap/pycmap/archive/master.zip) . Scripts central to the findings and analysis done in the manuscript are included in the Supplementary Information.

For manuscripts utilizing custom algorithms or software that are central to the research but not yet described in published literature, software must be made available to editors and reviewers. We strongly encourage code deposition in a community repository (e.g. GitHub). See the Nature Portfolio guidelines for submitting code & software for further information.

Data

Policy information about availability of data

All manuscripts must include a data availability statement. This statement should provide the following information, where applicable:

- Accession codes, unique identifiers, or web links for publicly available datasets
- A description of any restrictions on data availability
- For clinical datasets or third party data, please ensure that the statement adheres to our policy

All data used in this study is publicly available through the Simons Foundation CMAP (http://www.simonscmap.com, pycmap API available at https://github.com/simonscmap/pycmap/archive/master.zip), the listed resources in the Methods section and the Supplemental Information. Data downloadable from the Simons CMAP project using the pycmap API include: the Flombaum dataset (the original dataset from which the Flombaum model was created); the Atlantic Meridional Transect; the SeaFlow dataset; the GHRSST Level 4 AVHRR_OI Global Blended Sea Surface Temperature Analysis (GDS version 2) from NCEI, and the PAR data set

(MODIS PAR dataset). The full SeaFlow abundance and temperature data set external to CMAP is DOI: 10.5281/zenodo.3994953, direct download link from zenodo https://zenodo.org/record/3994953/files/SeaFlow_allstats_v.13_2020-08-21.zip?download=1. The HOT dataset was downloaded from Hawaii Ocean Time-series Data Organization & Graphical System (data from http://hahana.soest.hawaii.edu/hot/hot-dogs/). References in the accompanying 'Data Availability Statement' in the main text.

Field-specific reporting

Please select the one below that is the best fit for your research. If you are not sure, read the appropriate sections before making your selection.

☐ Life sciences ☐ Behavioural & social sciences ☒ Ecological, evolutionary & environmental sciences

For a reference copy of the document with all sections, see nature.com/documents/nr-reporting-summary-flat.pdf

Ecological, evolutionary & environmental sciences study design

All studies must disclose on these points even when the disclosure is negative.

Study description	This study tests the applicability of machine learning methods by focusing on the predictions of the 'Flombaum Model' for Prochlorococcus populations in a warming climate. The model and its dependent variables are tested as a function of spatial-temporal scale using previously published datasets.
Research sample	The sample consists of thousands of measurements of the abundance of Prochlorococcus, temperature, and photosynthetically active radiation (PAR). The sample was chosen to test previously developed algorithms. The original sample used to develop these algorithms was used, along with additional data used to test temporal dynamics (HOT ALOHA timeseries), and the effect of higher spatial resolution (SeaFlow Dataset).
Sampling strategy	Our manuscript explores the power of a previously published class of algorithm to predict Prochlorococcus abundance. We use more data than the original study, so our sample size is sufficient for our purposes. To simplify the interpretation and comparision of datasets we kept only samples which were in the layer of the ocean (here pre-defined as the upper 50 meters).
Data collection	Data for the various analysis was downloaded by VB and MC from the CMAP database. The data for the scaling analysis was downloaded directly (included in the Supplement) by CLF.
Timing and spatial scale	The datasets span 30 years of time and are global in scope. The Flombaum dataset referenced in the manuscript has a collection window of 1987-09-17 to 2008-11-10 . The SeaFlow Dataset on CMAP has a temporal window of 2010-05-04 to 2021-12-30.
Data exclusions	In order to simplify the analysis and interpretation, observations below the surface mixed layer of the ocean (>50 meters depth) were excluded from our analysis. This choice was made before the study started. Additionally, we wanted to use SeaFlow data (which is only surface) and satellite data (also surface) as part of our analysis.
Reproducibility	N/A no experiments were conducted for this manuscript
Randomization	We grouped the Flombaum dataset separately to do a direct comparision with the original algorithm. SeaFlow data was grouped for the scaling analysis due to its high spatial resolution. The HOT time series was grouped for the temporal analysis.
Blinding	Since the data was pre-existing in the literature, and the criteria for sub-sampling predetermined and quantitative, we did not use blinding in the data collation process.

Did the study involve field work? ☐ Yes ☒ No

Reporting for specific materials, systems and methods

We require information from authors about some types of materials, experimental systems and methods used in many studies. Here, indicate whether each material, system or method listed is relevant to your study. If you are not sure if a list item applies to your research, read the appropriate section before selecting a response.

Materials & experimental systems

n/a	Involved in the study
☒	☐ Antibodies
☒	☐ Eukaryotic cell lines
☒	☐ Palaeontology and archaeology
☒	☐ Animals and other organisms
☒	☐ Human research participants
☒	☐ Clinical data
☒	☐ Dual use research of concern

Methods

n/a	Involved in the study
☒	☐ ChIP-seq
☒	☐ Flow cytometry
☒	☐ MRI-based neuroimaging
